# Supplementary material for: Associations between EEG power and coherence with cognition and early precursors of speech and language development across the first months of life
Source: PLoS One. 2024 Apr 16;19(4):e0300382. doi: 10.1371/journal.pone.0300382 (PMC11020796; doi:10.1371/journal.pone.0300382)
Supplement: Attachment — Submitted filename: Revision_PlosOne_D-23-36319.pdf [file pone.0300382.s001.pdf]

The present study complements existing work with the aim of researching the early precursors of speech and language development across the first months of life through the analysis of EEG power and coherence in two significant frequency bands (3-6 Hz, Theta and 6-9Hz, Alpha).

I only have one concern regarding the way the work was conducted and was described in the preprocessing phase of the EEG data. There are some aspects on which I invite the authors to reflect.

In the paragraph 2.3 it is written that you use ica for removing artifacts. Several things have not been specified in this regard: the type of algorithm used, the length in time of the EEG signal to which you apply the IC, the number of components into which the EEG signal is decomposed and finally the sampling frequency of the acquired signal. This is all useful information to understand if the ICA has been used correctly since for example if the recorded signal is too short and was acquired with a sampling frequency that is not too high (below 1024 Hz) it is not It is always possible to break down the signal into a number of components equal to the number of electrodes because the information content is not sufficient (the number of points in which the signal was sampled for the length of the signal in time). The risk of using IC in an improper way is that mixed components are removed, i.e. those that contain both artefactual and cerebral signals because the software has not been able to separate the components adequately. Furthermore, information was omitted regarding the classification method of the artefactual components: manual or automatic? It should be noted that the most common brain signal artifact removal systems used on adult EEG signals are not always effective on children's EEG signals, especially on brain signals from children in their first year of age.
